# Identification of novel molecular subtypes to improve the classification framework of nasopharyngeal carcinoma

**DOI:** 10.1038/s41416-024-02579-w

**Published:** 2024-01-27

**Authors:** Wanzun Lin, Xiaochuan Chen, Zongwei Huang, Qin Ding, Hanxuan Yang, Ying Li, Duo Lin, Jun Lin, Haojiong Zhang, Xuelian Yang, Chao Li, Chuanben Chen, Sufang Qiu

**Affiliations:** 1 Department of Radiation Oncology, Clinical Oncology School of Fujian Medical University, Fujian Cancer Hospital, Fuzhou, China; 2 Fujian Provincial Key Laboratory of Translational Cancer Medicine, Fuzhou, China; 3 Ruijin Hospital, Shanghai Jiaotong University School of Medicine, Shanghai, China; 4 Key Laboratory of OptoElectronic Science and Technology for Medicine, Ministry of Education, Fujian Provincial Key Laboratory for Photonics Technology, Fujian Normal University, Fuzhou, China; 5 Institute of Apply Genomics, Fuzhou University, Fuzhou, China; 6 Shanghai Proton and Heavy Ion Center, Fudan University Cancer Hospital, Shanghai, China; 7 Department of Radiation Oncology, Longyan First Affiliated Hospital of Fujian Medical University, Longyan, China; 8 Department of Radiation Oncology, Second Hospital of Sanming City, Sangming, China

**Keywords:** Head and neck cancer, Tumour biomarkers

## Abstract

**Background:**

Nasopharyngeal carcinoma (NPC) treatment is largely based on a ‘one-drug-fits-all’ strategy in patients with similar pathological characteristics. However, given its biological heterogeneity, patients at the same clinical stage or similar therapies exhibit significant clinical differences. Thus, novel molecular subgroups based on these characteristics may better therapeutic outcomes.

**Methods:**

Herein, 192 treatment-naïve NPC samples with corresponding clinicopathological information were obtained from Fujian Cancer Hospital between January 2015 and January 2018. The gene expression profiles of the samples were obtained by RNA sequencing. Molecular subtypes were identified by consensus clustering. External NPC cohorts were used as the validation sets.

**Results:**

Patients with NPC were classified into immune, metabolic, and proliferative molecular subtypes with distinct clinical features. Additionally, this classification was repeatable and predictable as validated by the external NPC cohorts. Metabolomics has shown that arachidonic acid metabolites were associated with NPC malignancy. We also identified several key genes in each subtype using a weighted correlation network analysis. Furthermore, a prognostic risk model based on these key genes was developed and was significantly associated with disease-free survival (hazard ratio, 1.11; 95% CI, 1.07–1.16; *P* < 0.0001), which was further validated by an external NPC cohort (hazard ratio, 7.71; 95% CI, 1.39–42.73; *P* < 0.0001). Moreover, the 1-, 3-, and 5-year areas under the curve were 0.84 (95% CI, 0.74–0.94), 0.81 (95% CI, 0.73–0.89), and 0.82 (95% CI, 0.73–0.90), respectively, demonstrating a high predictive value.

**Conclusions:**

Overall, we defined a novel classification of nasopharyngeal carcinoma (immune, metabolism, and proliferation subtypes). Among these subtypes, metabolism and proliferation subtypes were associated with advanced stage and poor prognosis of NPC patients, whereas the immune subtype was linked to early stage and favorable prognosis.

## Background

Nasopharyngeal carcinoma (NPC) is a type of cancer that primarily affects people in Southeast Asia, especially South China [1, 2]. Distant metastasis and recurrence are the leading causes of death among patients with NPC and affect the improvement in the NPC cure rate in clinical practice [3]. Although the tumor-node-metastasis (TNM) staging technique is widely used in clinical settings, the anatomy-based staging process cannot efficiently predict patient prognosis or treatment findings. This results in uncertain heterogeneity in the clinical outcomes of NPC when using the same TNM staging approach [4–6]. Therefore, identifying the exact molecular subtypes that represent tumor heterogeneity is critical for predicting outcomes and tailoring specific treatment strategies for patients with NPC.

According to the prevailing World Health Organization (WHO), NPC is categorized into four primary histological subtypes: keratinizing (WHO type I), non-keratinizing differentiated (WHO type II), non-keratinizing undifferentiated (WHO type III), and basaloid squamous cell carcinoma [7, 8]. While the WHO subtype system remains the most commonly employed clinical classification for NPC, an increasing number of clinicians are recognizing its inadequacy in predicting the outcomes of chemotherapy and radiotherapy. The introduction and availability of next-generation sequencing tools have resulted in a large-scale data profile of many malignancies, allowing researchers to accurately characterize the tumors in a more structured manner [9, 10]. Because molecular events influence patient prognosis and therapeutic regimens, it is critical to determine and characterize the molecular subtypes of individual tumors. In NPC, although three molecular subgroups (immunogenic, classical, and mesenchymal) have been reported, this classification is based on miRNA expression, and differential pathway enrichment is indistinct among these subtypes [11]. Additionally, an epigenomic landscape study revealed global hypermethylation and hypomethylation in molecular subtypes; however, the sample size was limited [12]. The Cancer Genome Atlas recently defined the genomic landscapes and transcriptome subtypes of multiple malignancies, but not NPC [13]. A thorough understanding of NPC heterogeneity is lacking, and the translation of transcriptome outcomes into improved NPC treatments is required.

This study aimed to (i) profile gene expression in patients with NPC, (ii) identify novel molecular subtypes to improve the classification of NPC, and (iii) analyze the clinicopathological features of these subtypes.

## Methods

### Clinical sample collection

Fresh tumor biopsy samples were collected from patients diagnosed with NPC at Fujian Cancer Hospital between January 2015 and January 2018. TNM staging was used to confirm and classify all collected tumor samples. Table 1 presents the clinical data of the patients with NPC. This study was approved by the Ethics Committee of Fujian Cancer Hospital (Fuzhou, China; numbers K2022-084-01). Each patient provided written informed consent for participation in this study. The external validation cohorts were downloaded from the Gene Expression Omnibus (GEO) database, including GSE102349 and GSE103611 datasets. RNA expression files of NPC cell lines (NP69, HK-1, CNE-2, and 5–8F) were derived from GSE15098, GSE29123 and GSE15921.

**Table 1:** Clinical characteristics of NPC patients.

Characteristics	*n* = 192
Age	48 (16–87)
Sex	
Female	56 (29.2%)
Male	136 (70.8%)
T-category, 7th	
T1	40 (20.8%)
T2	43 (22.4%)
T3	62 (32.3%)
T4	47 (24.5%)
N-category, 7th	
N0	14 (7.3%)
N1	67 (34.9%)
N2	79 (41.1%)
N3	32 (16.7%)
TNM stage, 7th	
I	4 (2.1%)
II	40 (20.8%)
III	73 (38.0%)
IVa	48 (25.0%)
IVb	19 (9.90%)
IVc	8 (4.2%)

### RNA isolation, mRNA library construction, and sequencing

A TRIzol reagent kit was used to extract total RNA according to the manufacturer’s instructions. Furthermore, mRNA samples from NPC tissues were enriched using oligo (dT) -attached magnetic beads. The enriched mRNA samples were then separated into fragments using a fragmentation buffer, followed by reverse transcription into cDNA. Then, a single ‘A’ nucleotide was added to the 3’ ends of the blunt fragments after repairing the ends of the purified double-stranded cDNA segments. The reaction system and program for adapter ligation were configured to ligate the adapters with cDNAs, and PCR was performed to amplify them. The generated cDNA library was sequenced by BGI Technology Services Co. Ltd.

### Extraction of all metabolites and LC-MS/MS analysis

We collected 1 × 10^7^ cells in a clean tube. Then, extraction solution (acetonitrile: methanol = 1:1, with an isotopically-labeled internal standard mixture) was added to the sample, and the mixture was vortex-mixed for 30 s, sonicated for 10 min in an ice-water bath, and incubated at −40 °C for 60 min for precipitating the proteins. Then, this mixture was centrifuged at 12,000 rpm for 15 min at 4 °C. For UHPLC-QE-MS analysis, the supernatant was transferred to a different LC-MS glass vial (2 mL). Finally, a quality control sample was prepared by mixing equal aliquots of the supernatants from all samples. A UHPLC system (Vanquish, Thermo Fisher Scientific, USA) that included a UPLC BEH Amide column coupled to a Q Exactive HFX mass spectrometer (Orbitrap MS, Thermo Scientific) was used for the LC-MS/MS analysis. The variable importance in the projection (VIP) score of the (O)PLS model was used to rank the metabolites that best distinguished the two groups. The VIP threshold was set at 1. Univariate analysis was performed using the *t*-test to analyze different metabolites. The metabolites that showed a | foldchange| ≥ 2 and *p*-value of *t*-test <0.05 were regarded as differential metabolites between both groups.

### Gene set variation analysis

Gene Set Variation Analysis (GSVA) was performed to obtain the pathway score of every NPC sample based on transcriptome data, using the “GSVA” package in R software (version 4.2.1). GSVA computes the enrichment score for each sample by evaluating the expression levels of genes within a predefined gene set. This score serves as a representative measure of the overall activity or enrichment of a specified gene set within a sample [14, 15]. In our analysis, KEGG gene sets were used to obtain a finer resolution of functional signature variations across samples.

### Consensus clustering

The “ConcensusClusterPlus” tool in R software was used to identify the molecular subtypes by means of consensus clustering. Thereafter, the optimal cluster value between *k* = 2 and 10 was determined, and this technique was performed 1000 times to ensure that all results were reproducible and robust.

### Principal component analysis

Principal component analysis (PCA) was performed to evaluate the transcriptional patterns of different subtypes. The analysis was conducted using the “princomp” function in the “limma” package, and these results were displayed using the “ggplot2” package in R.

### Weighted gene co-expression network analysis

Weighted gene co-expression network analysis (WGCNA) was conducted using the R software (version 4.2.1) with default parameters [16–18]. The Pearson correlation between each module and each phenotypic characteristic within each subtype (immune, metabolic, and proliferative) was derived and corrected using the Benjamini–Hochberg FDR method. Within each module, the hub genes were identified based on their high connectivity (correlation) with other genes in the module. These hub genes are considered as key regulators within the module.

### Immune cell type fractions analysis

The Tumor Immune Estimation Resource (TIMER) algorithm was applied to calculate the infiltration levels of eight types of immune cells in each NPC sample, including macrophages, B cells, CD4 T cells, CD8 T cells, neutrophils, and dendritic cells (DCs). TIMER employs algorithms to perform deconvolution and estimate the proportion of immune cells in tumor samples [19, 20].

### Immune score estimation

The immune score and tumor purity were calculated using the estimation of stromal and immune cells in malignant tumor tissues using expression data (ESTIMATE). The ESTIMATE algorithm, implemented in R software (version 4.2.1), was designed to estimate the fractions of stromal, tumor, and immune cells in tumor tissues based on RNA sequencing data. Specifically, it uses a predefined set of genes expressed by immune cells, and the expression levels of these genes in the tumor sample are used to infer the abundance of immune cells in the tumor microenvironment.

### Constructing and validating the prognostic risk signature

The coefficient values were then computed using the least absolute shrinkage and selection operator (LASSO) Cox regression. LASSO, a regression analysis technique, conducts variable selection and regularization to enhance both the predictive accuracy and interpretability of a statistical model. Consequently, LASSO Cox regression is well-suited for constructing prognostic models grounded in gene expression profiles [21–25]. Kaplan–Meier (KM) analysis was then performed to compare OS between the low- and high-risk groups. The Survminer and survival packages of R software were used for KM analysis. The NPC dataset (GSE102349) was used as the validation set [26].

### Assessment of cytotoxic activity of compounds using CCK-8 assay

NPC cell lines HK1, CNE-2, and 5–8F were seeded in 96-well plates at a density of 1 × 10^5^, or 4 × 10^4^cells per well and cultured overnight at 37 °C and allowed to attach for 24 h. Indomethacin was added at graded concentrations with 0, 100, 250, 400, 500, and 1000 µg/mL incubated for 72 h. Cell viability was evaluated using the CCK-8 assay in accordance with the manufacturer’s guidelines. Dose-response curves were generated, and the 50% inhibition concentration (IC50) was determined, representing the concentration of compounds that led to a 50% reduction in cell growth compared to the control.

### Cell proliferation assays

The EdU assay was conducted to investigate the impact of arachidonic acid on the proliferation of NPC cell lines. HK1, CNE-2, and 5–8F cells were seeded in a 24-well plate at a density of 2 × 10^5^ cells per well and allowed to incubate overnight. Subsequently, a conditioned medium containing indomethacin at various IC50 concentrations (450, 300, 250 µg/mL) was applied for 24 h at 37 °C with 5% CO2. Following this, 100 µL of EdU (50 µM) was introduced to the culture medium for an 8-h incubation period. The cells were then fixed with 4% paraformaldehyde for 20 min, permeabilized using Triton X-100 to breach the nuclear membrane, and blocked with PBS for an additional 1 h at 25 °C. Finally, cell staining was performed using the BeyoClick™ EdU-555 Cell Proliferation Kit (Beyotime, China) in accordance with the manufacturer’s instructions. A negative control was established using 1% DMSO.

### Cell migration assay

The migration assay was conducted using a 24-well plate with 8.0 μm pore membrane inserts (Corning, NY) devoid of matrigel. NPC cell lines were introduced into the upper chambers at a concentration of 1 × 105 cells per well and incubated for 24 h, followed by treatment with various IC50 concentrations of indomethacin. The upper chambers were maintained in a conditioned medium without serum, while the lower chambers were filled with conditioned media containing 20% serum. After 24 h (5–8F cells) or 36 h (CNE-2 cells), the migrated cells were stained with 0.1% crystal violet and photographed under a light microscope at 100× magnification. For HK1 cells, migration was observed over a period of 5 days. The number of migratory cells was quantified and subjected to statistical analysis to ascertain significant differences. The migration assay was independently replicated three times, with 1% DMSO serving as the negative control.

### Statistical analysis

Statistical data analysis was conducted using GraphPad Prism 8.4.1. For the comparison of two groups, a two-tailed unpaired Student’s *t*-test or the Wilcoxon test was employed. In scenarios involving more than two groups, the Kruskal–Wallis test was applied for comparison.

## Results

### Consensus clustering identified immune, metabolism, and proliferation subtypes with distinct clinical features in patients with NPC

Transcriptomic analysis was performed on 192 treatment-naïve primary NPC acquired from the Fujian Cancer Hospital. Table 1 summarizes the clinicopathological information of the patients, including sex, age, T-category, N-category, and TNM stage as described by the American Joint Committee on Cancer. This dataset comprised patients with a median age of 48 years, and included 29.2% (*n* = 56) female patients and 70.8% (*n* = 136) male patients. Furthermore, 44 (22.9%) patients developed stage I/II cancer, 73 (38.0%) developed stage III tumors, and 75 (39.1%) developed stage IV tumors.

**Fig. 1:**
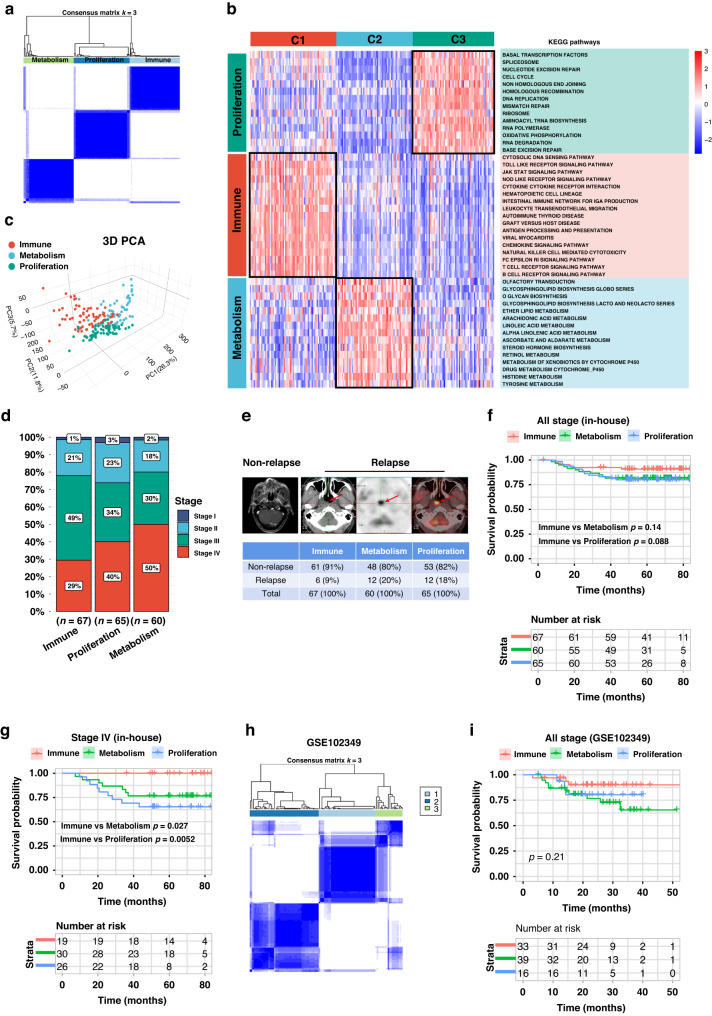
Consensus clustering identified three molecular subtypes in patients with NPC. **a** Heatmap of consensus clustering solution (*k* = 3) in 192 NPC samples. **b** Heatmap of pathway score in the proliferation, immune and metabolism molecular subtypes (*n* = 192). **c** Principal component analysis plots revealing distinct expression patterns among three subtypes; red dots represent immune subtype, blue dots represent metabolism subtype, and green dots represent proliferation subtype. **d** Bar plots presenting the frequency of different stages among these subtypes. **e** Relapse rate among these subtypes. NPC relapse was presented by PET/CT (positron emission tomography/computed tomography). **f**, **g** Kaplan–Meier disease-free survival curve for all patients with NPC (F) or stage IV patients (**g**) assigned to immune, metabolism, and proliferation subtypes in the in-house cohort. **h** Validation of three molecular subtypes classification in GSE102349 external cohort. **i** Validation of Kaplan–Meier analysis in GSE102349 external cohort.

RNA sequencing was performed to obtain gene expression profiles. To quantify the pathway activity of each NPC sample, pathway signatures were acquired from the Kyoto Encyclopedia of Genes and Genomes database. Thereafter, the GSVA was used to determine the pathway score based on transcriptomics. Subsequently, NPC subtypes were identified using consensus clustering. The k-means clustering technique was used and three clusters were identified to describe the different patterns of pathway activity (Fig. 1a). Specifically, cluster C1 showed high activity in immune pathways, such as natural killer cell-mediated cytotoxicity, antigen processing and presentation, T-cell receptor signaling pathway, leukocyte transendothelial migration, and chemokine signaling pathways (Fig. 1b). In contrast, clusters C2 and C3 displayed low immune pathway activity, but high metabolic activation (e.g., histidine metabolism, arachidonic acid metabolism, ether lipid metabolism, and retinol metabolism) and proliferation pathways (e.g., cell cycle, DNA replication, mismatch repair, ribosome, and homologous recombination). Hence, the C1 cluster was defined as the immune subtype, the C2 cluster was defined as the metabolic subtype, and the C3 cluster was defined as the proliferative subtype (Fig. 1b). PCA technique was used to compare transcriptional patterns among different subtypes. In general, PCA results indicated that the samples from the three clusters were strongly isolated from each other, indicating that these subtypes displayed different transcriptional profiles and heterogeneity (Fig. 1c).

Next, we explored whether these three subtypes were associated with different clinical features. Intriguingly, patients classified into immune subtypes had an early clinical stage, low relapse rate, and favorable long-term prognosis. Conversely, the metabolism and proliferation subtypes primarily included advanced clinical stages, high relapse rates, and poor prognoses (Fig. 1d–f). Subgroup analysis of stage IV patients demonstrated statistically significant differences in disease-free survival DFS (Fig. 1g). The repeatability of this classification was verified in two independent public cohorts (GSE102349, *n* = 88; GSE103611, *n* = 48). Similarly, patients in the GSE102349 (Fig. 1h, i) and GSE103611 cohort (Fig. S1A, S1B) were stratified into immune, metabolic, and proliferative subtypes.

### Immune, metabolism, and proliferation subtypes are associated with distinct tumor microenvironments

**Fig. 2:**
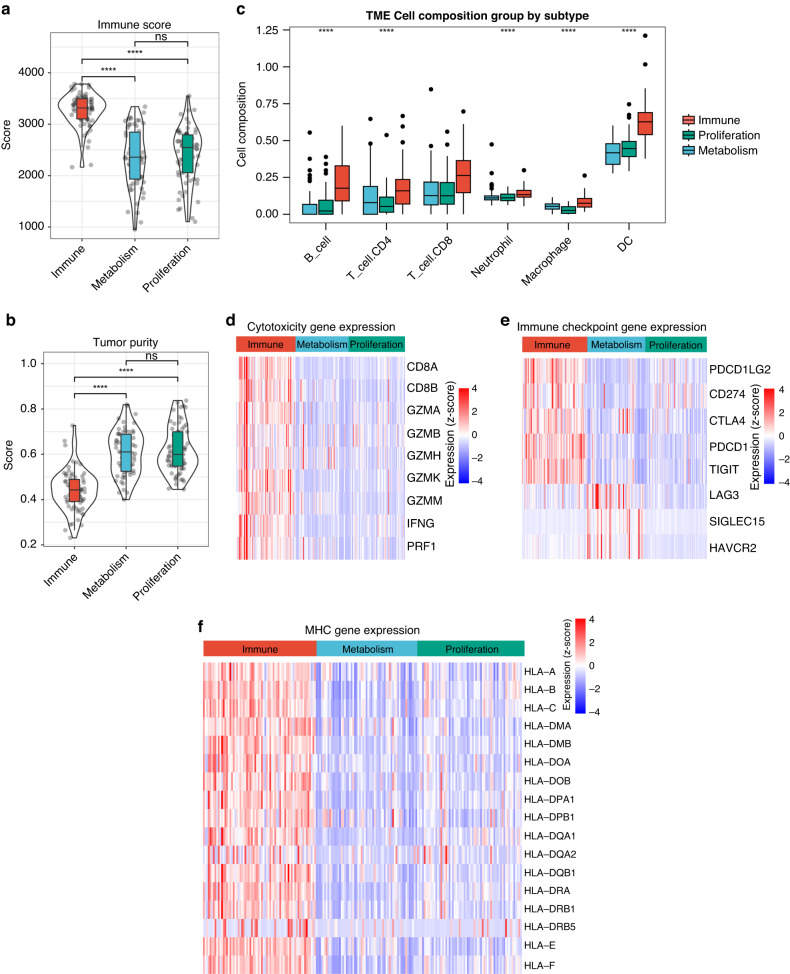
Three subtypes are associated with distinct tumor microenvironments. Violin plots showing the median, quartile, and kernel density estimations for each immune score (**a**) and tumor purity score (**b**). **c** Box plot of 6 immune cell population score among three subtypes. Red boxes represent immune subtype, blue boxes represent metabolism subtype, and green boxes represent proliferation subtype. The upper, middle, and lower horizontal lines of the box represent the upper, median, and lower quartile respectively. Heatmaps presenting the differential cytotoxicity gene expression (**d**), immune checkpoint gene expression (**e**), and MHC gene expression (**f**) among the three subtypes; the expression values were adjusted by z-score. *P*-values were determined using the Wilcoxon or Kruskal–Wallis tests (ns, *p* > 0.05; ****, *p* < 0.001).

In this study, we analyzed the composition of the tumor microenvironments of the different subtypes. In general, the immune subtype displayed a higher immune score than the proliferation and metabolism subtypes in the in-house cohort and external validation cohorts (Figs. 2a,  S2A), whereas tumor purity showed a lower score (Figs. 2b, S2B). The TIMER method was used to assess immune heterogeneity across different subtypes. Patients with the immune subtype had a significantly higher proportion of B cells, CD8+ T cells, CD4+ T cells, macrophages, neutrophils, and DCs than did those with the metabolic and proliferative subtypes (Figs. 2c, S2C). Furthermore, the majority of immune checkpoints, MHC genes, and cytotoxicity genes were overexpressed in the immune subtype. The metabolic and proliferative subtypes showed opposite trends (Fig. 2d–f). These findings suggest that patients with immune subtypes may benefit from immune checkpoint inhibitor therapy.

### Identification of the key metabolites in metabolism subtype

**Fig. 3:**
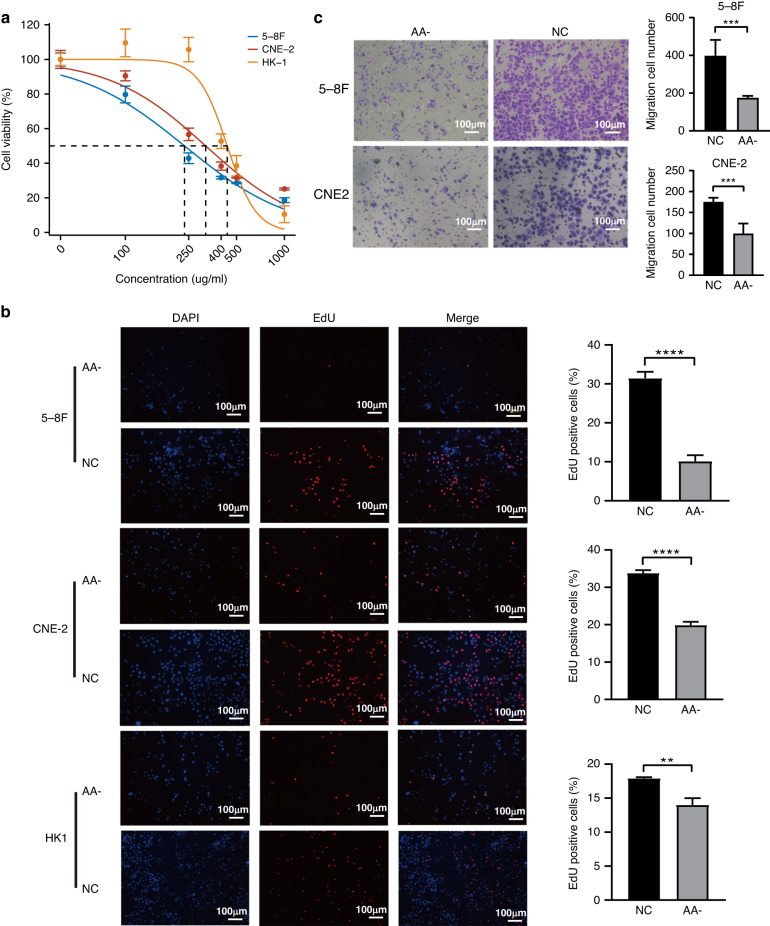
Arachidonic acid inhibitor suppressed NPC cells proliferation and migration. **a** Cell viability determined by CCK-8 analysis. **b** EdU assay was carried out to investigate the proliferation in NPC cell lines. Cell nuclei stained in red represent DNA replication. NC, control; AA-, arachidonic acid inhibition. Scale bar, 100 μm. **c** Effects of arachidonic acid inhibitor on the migration of NPC cell lines by transwell assays. Scale bar, 100 μm. *P*-values were determined via Student’s *t*-test (**, *p* < 0.01; ***, *p* < 0.001; ****, *p* < 0.0001).

Metabolic reprogramming is a characteristic of cancer and an essential target for cancer therapy. We initially classified NPC cell lines (CNE-2, 5–8F, HK-1, and NP69) based on their RNA-seq expression profiles. Notably, the CNE-2 and 5–8F cell lines exhibited higher metabolic pathway activity, whereas NP69 and HK-1 showed lower metabolic pathway activity (Fig. S3A). To further verify the key metabolites that drive the malignant phenotype of NPC, a metabolomic analysis was performed between CNE-2 and NP69 cells. In total, 27 differentially expressed metabolites were identified under negative-ion mode with |fold change| > 2 and *p* < 0.05 (Fig. S3B). We found arachidonic acid was obviously up-regulated in CNE-2 compared to NP69 cell line, and arachidonic acid metabolism pathway was significantly enriched in NPC metabolism subtype (Fig. S3C). Next, we conducted a series of in vitro experiments to preliminarily validate arachidonic acid metabolism in NPC. We found that inhibition of arachidonic acid metabolism by indomethacin significantly reduced the viability of HK1, CNE-2, and 5–8F cells in a concentration-dependent manner, with IC50 values of 436.7 μg/mL, 319.6 μg/mL, and 235.1 μg/mL, respectively (Fig. 3a). Interestingly, compared to HK-1 cell line, CNE-2 and 5–8F exhibited higher sensitivity to indomethacin, possibly due to the elevated activity of the arachidonic acid metabolism pathway in these two cell lines. Next, we further investigated whether inhibition of arachidonic acid metabolism (AA-) could inhibit the malignancy of NPC cells. The results revealed that indomethacin could decrease the percentage of EdU-positive cells in HK1, CNE-2, and 5–8F cells (Fig. 3b). Moreover, transwell assays demonstrated arachidonic acid inhibition could significantly decelerate CNE-2 and 5–8F cell migration (Fig. 3c).

### Establishment and verification of the NPC prognostic signature

**Fig. 4:**
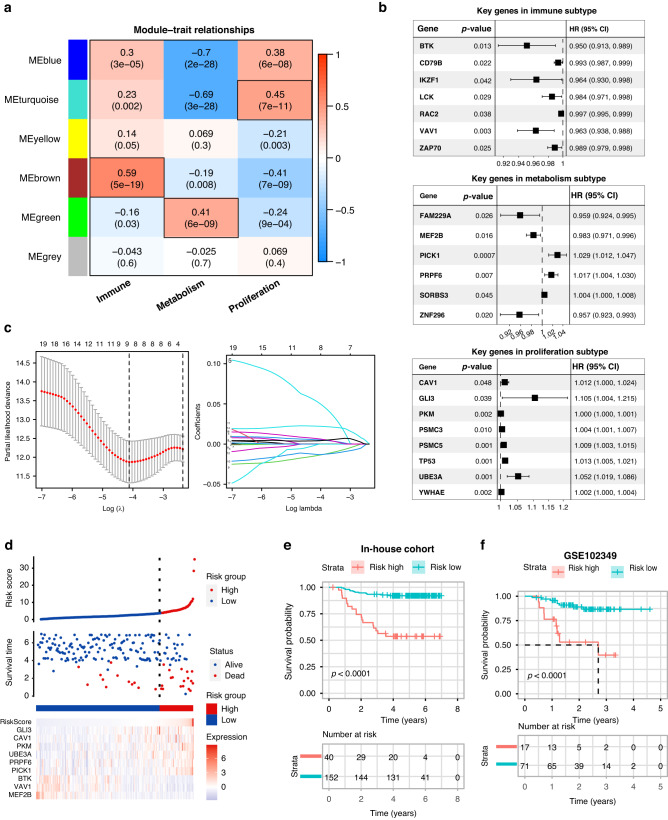
Establishment and verification of the NPC prognostic signature. **a** Heatmap of the correlation between module eigengenes and subtypes of NPC. Each table cell contains the correlation coefficient and *p*-value. The color shade represents the correlation coefficient and *p*-value is described in parenthesis. **b** Univariate Cox analysis of key genes identified by WGCNA in three subtypes. **c** Lasso Cox analysis uncovered nine genes most associated with DFS. The best parameter (*λ*) was selected based on the LASSO algorithm. **d** Risk scores distribution, survival status of each patient, and heatmap of nine prognostic risk genes expression. Kaplan–Meier curves for patients with high- or low-risk scores in the in-house training cohort (**e**) and GSE102349 testing cohort (**f**). DFS is selected as a statistical indicator.

We further developed a prognostic model based on the key genes of the three subtypes. To identify key genes in these subtypes, we applied WGCNA to the gene expression matrix. The characteristic genes of the brown, green, and turquoise modules were remarkably related to the immune, metabolic, and proliferative subtypes, respectively (Fig. 4a). We identified seven key genes with significant prognostic value in the brown module of the immune subtype, 6 key genes in the green module of the metabolic subtype, and eight key genes in the turquoise module of the proliferative subtype (Fig. 4b). As depicted in Fig. 4c, the 21 key genes identified by WGCNA were evaluated and selected to establish a prognostic model using LASSO regression analysis. Thereafter, a risk score model was developed using the following equation: Risk score = 0.00029 × PKM + 0.00526 × CAV1 + 0.05278 × GLI3 + 0.02225 × PICK1 + 0.00672 × PRPF6 + 0.00403 × UBE3A − 0.01432 × MEF2B − 0.01298 × VAV1 − 0.00076 × BTK. In this study, the relationship between the survival status and risk score was assessed. The results revealed that the number of live statuses of patients in the high-risk cohort was remarkably lower than that in the low-risk cohort (Fig. 4d). The prognostic significance of this risk model was further determined using KM analysis. A high-risk score was associated with poor DFS in the in-house training cohort (Fig. 4e), which was verified using the GEO testing cohort (Fig. 4f). In addition, we further demonstrated the prognostic value of risk model in the subgroup analysis of each subtype. The high-risk score was associated with unfavorable DFS in immune, metabolism, and proliferation subtypes (Fig. S4A).

### NPC risk signature exhibits a strong power for prognosis assessment

**Fig. 5:**
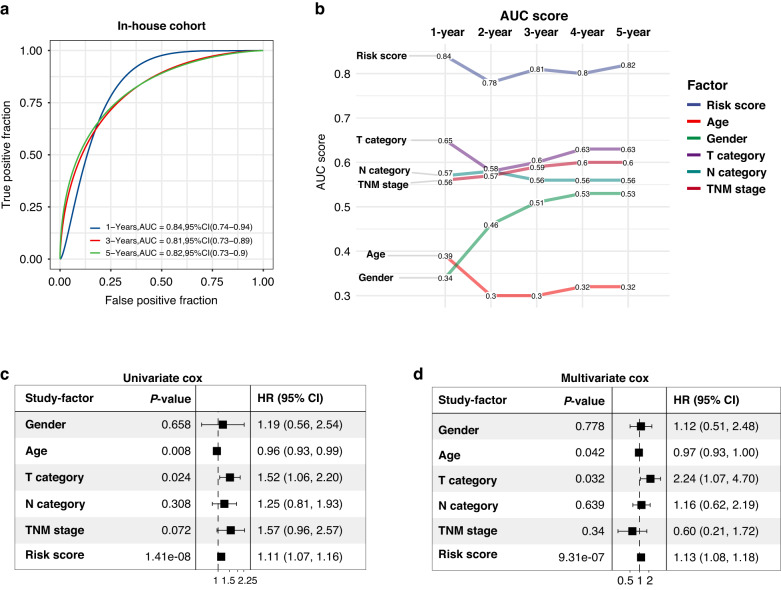
NPC risk signature exhibits a strong power for prognosis assessment. **a** ROC curve showing the predictive value of NPC risk signature for 1-, 3-, and 5-year survival rates. **b** Comparison of predictive value between NPC risk signature and clinicopathologic features. Univariate Cox (**c**) and multivariate Cox analyses (**d**) evaluating the independent prognostic value of the NPC risk signature in terms of DFS.

A receiver operating characteristic (ROC) curve was used to assess the predictive efficiency of the risk signature for the 1-, 3-, and 5-year survival rates. The areas under the ROC curve values were 0.84, 0.81, and 0.82, respectively, showing a high predictive value (Fig. 5a). We also compared the predictive efficiency of the NPC risk signature with clinical characteristics including age, sex, T-category, N-category, and clinical stage. The results demonstrated that the risk score presented the best performance in predicting prognosis compared to the other clinical characteristics (Fig. 5b).

The independent predictive value of the risk prognostic model for DFS was assessed using univariate and multivariate Cox regression analyses. Univariate analysis indicated that a high-risk score was significantly associated with poor DFS (Fig. 5c). Other variables associated with poor survival included age and T-category. Multivariate analysis revealed that a high-risk score was independently associated with a significantly worse DFS, suggesting that it could be employed as an independent prognostic factor for patients with NPC (Fig. 5d).

## Discussion

Precision oncology utilizes advanced molecular profiling techniques such as next-generation sequencing and gene expression analysis to identify specific genetic mutations or biomarkers in tumors. These techniques provide novel insights into cancer diagnostics and targeted therapeutics. In this study, we introduced a novel molecular classification of NPC based on transcriptomic analysis, namely immune, metabolic, and proliferative subtypes with distinctive clinical features. These results indicated that the immune subtype was associated with early clinical stages and a favorable long-term prognosis. Conversely, the metabolic and proliferative subtypes mainly include advanced clinical stages and poor prognoses. In addition, the findings of this study show that arachidonic acid metabolites and pathways are associated with NPC progression. The WGCNA technique was used to identify key genes among the subtypes. We also identified several key genes in each subtype using WGCNA. Moreover, we developed and validated a prognostic model based on these key genes, which has a strong potential for prognosis assessment.

The canonical histopathology of NPC includes keratinizing, non-keratinizing differentiated, non-keratinizing undifferentiated, and basaloid squamous cell carcinoma. However, even with the same histological type or TNM stage, patient responses to treatment and prognoses vary when identical therapeutic approaches are employed [27–29]. In recent years, with the advancement of sequencing technologies and the establishment of various platforms, such as high-throughput RNA sequencing, proteomics, metabolomics, and single-cell sequencing, our understanding of tumor heterogeneity has significantly improved. Molecular subtyping of NPC is a dynamic field of research, with several studies attempting to delineate its molecular intricacies. For example, genomic studies have identified genetic alterations in NPC, including loss of the CDKN2A/CDKN2B locus, CCND1 amplification, TP53 mutation, and activation of signaling pathways such as the PI3K/Akt/mTOR pathway, DNA repair, chromatin modification, and MAPK signaling [1, 26, 30–32]. For RNA expression studies, although microRNA sequence-based analysis was used to classify NPC into immunogenic, classical, and mesenchymal subtypes, the prognostic value of this classification is limited [11]. In addition, an immune-associated classification has been delineated, wherein the immune-enriched subtype manifests noteworthy enrichment of immune cells, whereas the non-immune subtype exhibits highly proliferative characteristics [33]. In the present study, an original RNA expression file was established from 192 patients with NPC. We further classified the non-immune subtypes into proliferative and metabolic subtypes. The results of this study showed that this classification system was repeatable and predictable and displayed a high prognosis evaluation value. Nevertheless, it should be noted that our findings require further internal cohort validation. Our findings should be interpreted with this limitation in mind.

We also identified a panel of key genes among these subtypes that were significantly associated with NPC prognosis. Among these genes, PKM, CAV1, GLI3, PICK1, PRPF6, and UBE3A have been identified as oncogenes, and play a pivotal role in orchestrating tumor-host interactions, fostering tumor growth, promoting metastasis, enhancing resistance to therapy, and ensuring cellular survival. For example, PKM serves as a pivotal enzyme in glycolysis and functions as a mediator of the Warburg effect observed in tumors [34]. CAV1 operates as a pro-survival factor, playing a role in mediating resistance against the cytotoxic effects induced by ionizing radiation [35]. Additionally, PRPF6, an integral component of the tri-snRNP (small ribonucleoprotein) spliceosome complex, propels cancer proliferation through its involvement in the preferential splicing of genes associated with cellular proliferation [36]. GLI3 and PICK1 were found to over-express in various cancers, including colorectal cancer, ovarian cancer, breast cancer, prostate cancer, and oral squamous cell carcinoma [37–40]. On the contrary to these oncogenes, MEF2B, VAV1, and BTK, negatively associated with risk score, have been identified as immune regulatory genes. VAV1 is a key player in the signaling cascades initiated by antigen receptors on lymphocytes. It is involved in the activation of T-cell and B-cell receptors, leading to immune responses [41, 42]. BTK is a critical component of the BCR signaling pathway, mediating B-cell activation, proliferation, and differentiation [43]. However, the functions of these key genes require further validation in vitro or in vivo.

Immunotherapy, particularly with immune checkpoint inhibitors (ICIs), is extremely effective for NPC treatment [44–46]. Except for a few intriguing clinical trials, the general application of immunotherapies has yielded different responses, with a proportion of patients displaying resistance to these treatments. Therefore, classifying patients with NPC with high or low sensitivity would assist in optimizing the effectiveness of immunotherapy. Previous studies identified several predictive biomarkers for immunotherapy, including tumor mutational burden, microsatellite instability, lymphocytes, immune score, T-cell receptor diversity, and PD-L1/PD1 expression [47–49]. However, the predictive value of single biomarkers is limited. In this study, an immune subtype was characterized by high PD-L1/PD1 expression, cytotoxicity genes, MHC genes, and high infiltration of immune cells. This finding indicates that patients with different immune subtypes may benefit from ICI therapy.

In conclusion, this study presented a novel classification of NPC based on transcriptomics. This classification demonstrated a significant value in predicting the survival in patients with NPC.

### Supplementary information

Supplementary Material

## Data Availability

All materials underlying this study are available from the corresponding author based on a material transfer agreement.

## Code availability

The codes used in this study have been reported previously and are available as described in the corresponding Methods section.
