# Supplementary material for: Identification of novel molecular subtypes to improve the classification framework of nasopharyngeal carcinoma
Source: Br J Cancer. 2024 Jan 27;130(7):1176–86. doi: 10.1038/s41416-024-02579-w (PMC10991292; doi:10.1038/s41416-024-02579-w)
Supplement: Supplementary file 1 — Supplementary Material [file 41416_2024_2579_MOESM1_ESM.doc]

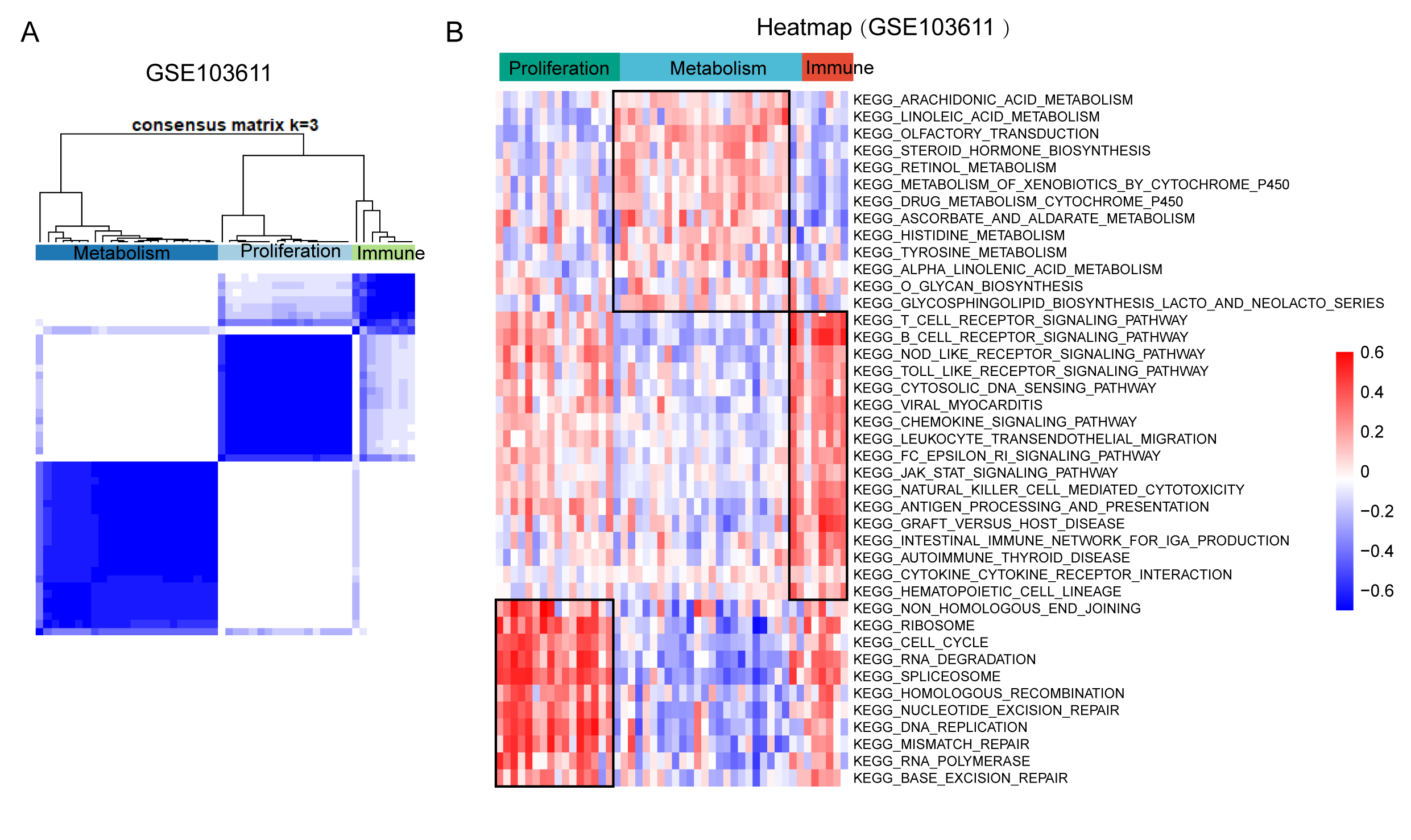


**Figure S1. (A)** Heatmap of consensus clustering solution (k = 3) in GSE103611 dataset. **(B)** Heatmap of pathway score in GSE103611 dataset.


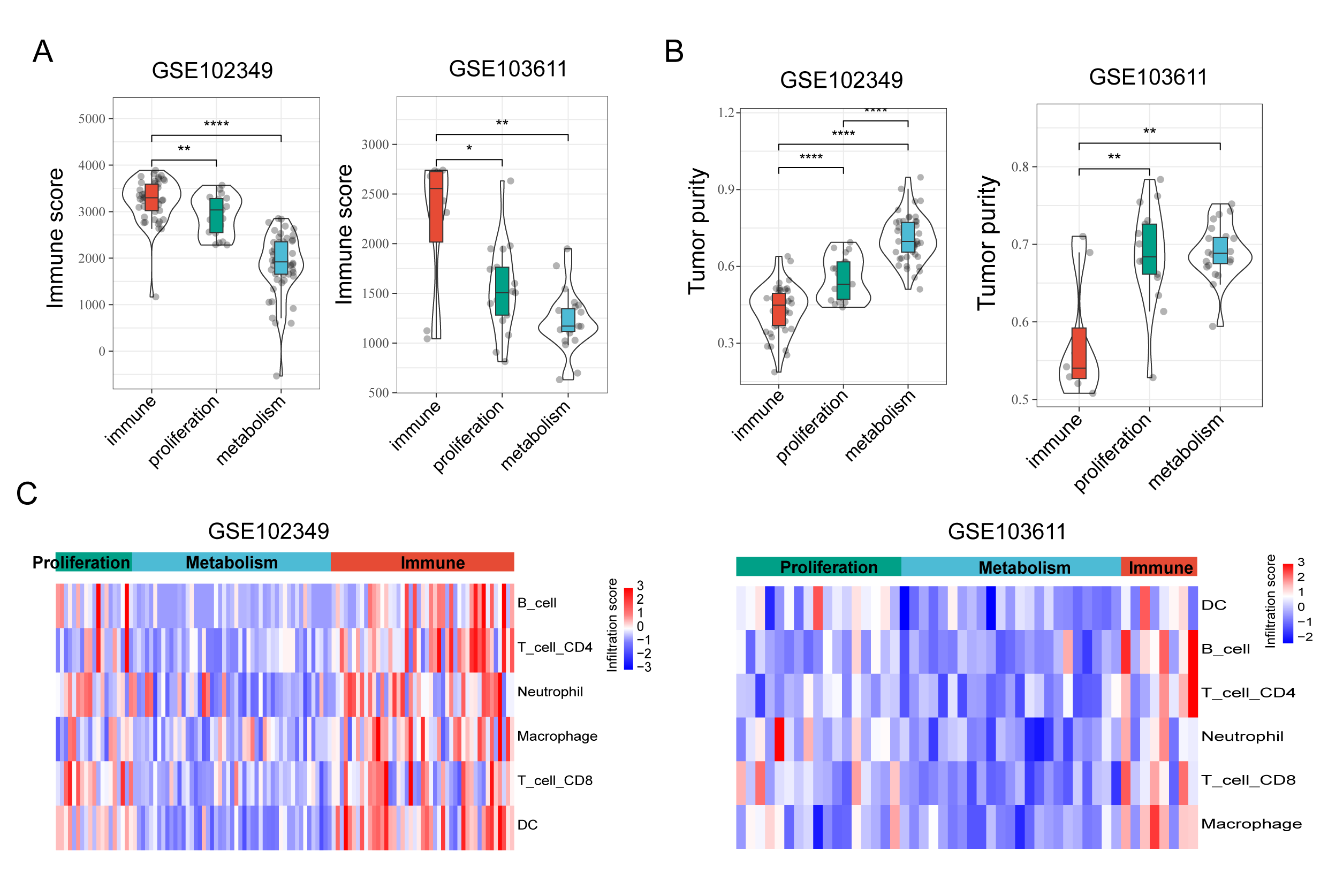


**Figure S2. (A, B)** Violin plots showing the median, quartile, and kernel density estimations for each immune score (A), and tumor purity score (B) in GSE102349 and GSE103611 validation datasets. **(C)** Heatmap of 6 immune cell population scores among three subtypes in validation datasets.


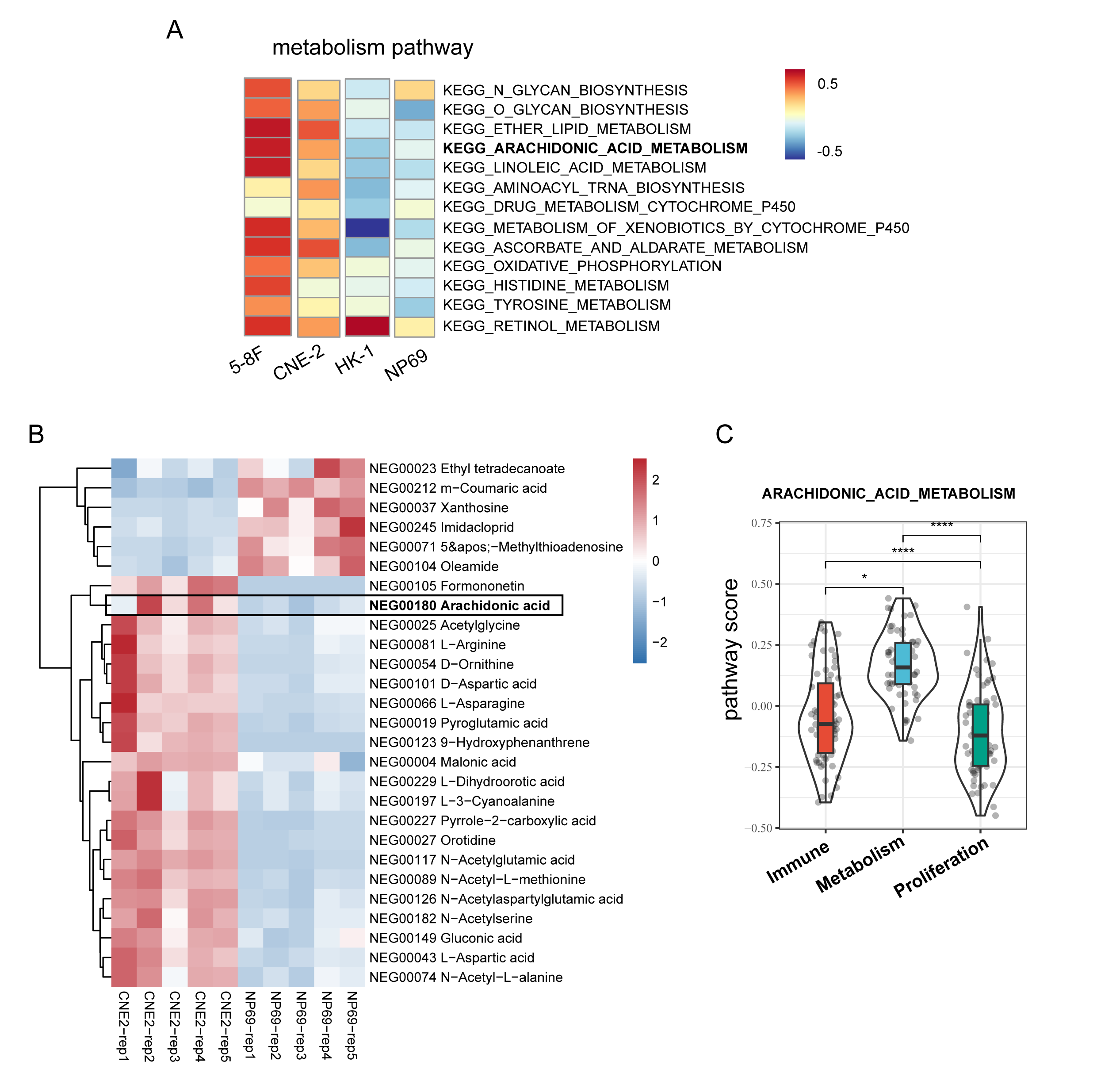


**Figure S3. (A)** Heatmap of metabolism pathway score in 5-8F, CNE-2, HK-1 and NP69 cell lines. Pathway score was calculated by GSVA method based on the RNA-seq expression profiles. **(B)** Heatmap of differential metabolites between CNE-2 and NP69 cells. Individual samples (horizontal axis) and compounds (vertical axis) are separated using hierarchical clustering. **(C)** Violin plots showing the ARACHIDONIC_ACID_METABOLISM pathway score in the immune, metabolism, and proliferation subtypes. using the Wilcoxon or Kruskal–Wallis tests (*, p<0.05; ****, p < 0.001).


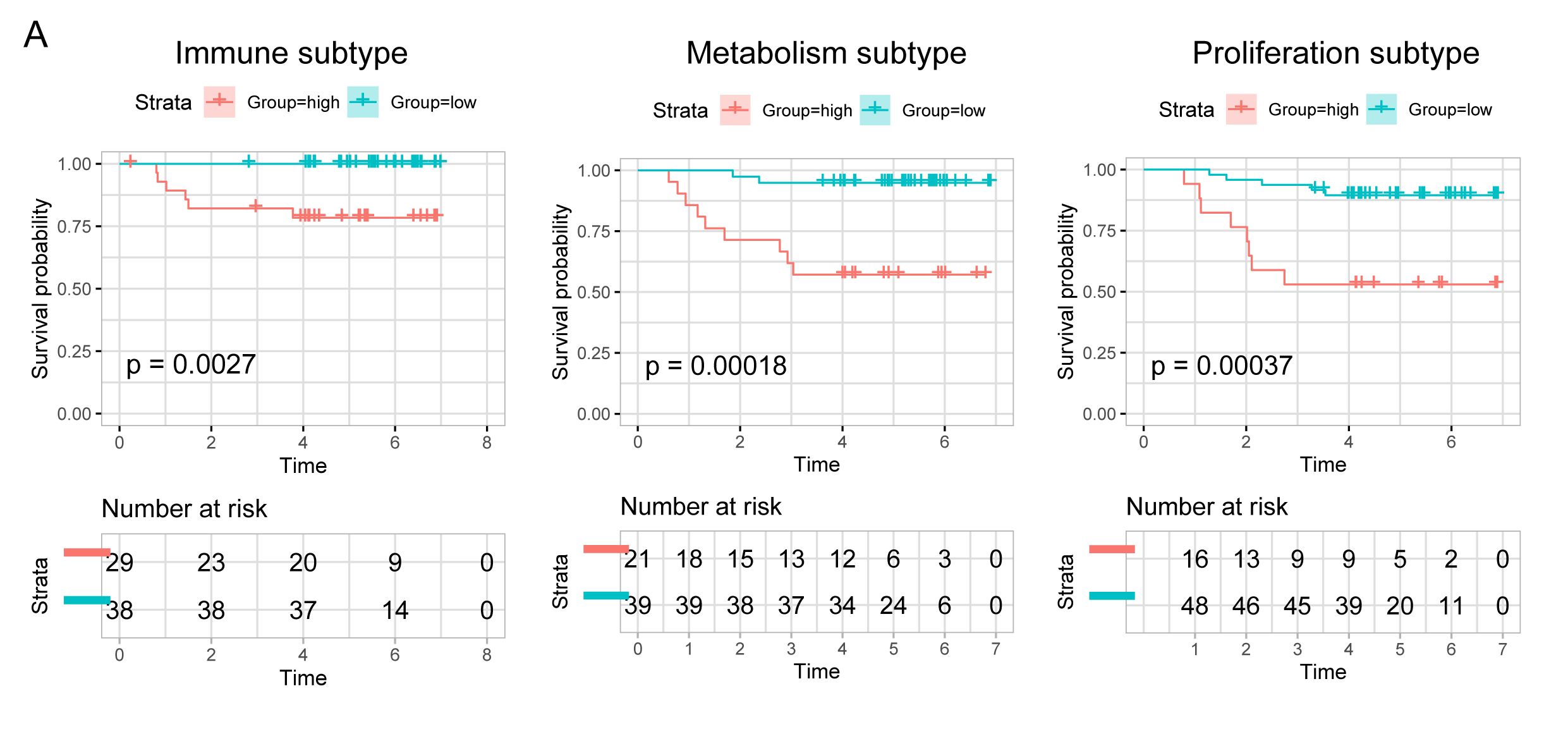


**Figure S4. (A)** The prognostic value of risk model in each subtype.
